# Association of Serum Total Bilirubin to Cholesterol Ratio With Progression of Chronic Kidney Disease in Patients With Type 2 Diabetes: A Retrospective Cohort Study

**DOI:** 10.1111/1753-0407.70097

**Published:** 2025-05-13

**Authors:** Yanyan Chen, Shanshan Wang, Hang Guo, Fei Han, Bei Sun, Nan Li, Hongxi Yang, Liming Chen

**Affiliations:** ^1^ NHC Key Laboratory of Hormones and Development, Tianjin Key Laboratory of Metabolic Diseases, Chu Hsien‐I Memorial Hospital & Tianjin Institute of Endocrinology Tianjin Medical University Tianjin China; ^2^ Research Center of Clinical Epidemiology Peking University Third Hospital Peking China; ^3^ Department of Bioinformatics, School of Basic Medical Sciences Tianjin Medical University Tianjin China

**Keywords:** chronic kidney disease, cohort study, renal outcome, total bilirubin to total cholesterol ratio, type 2 diabetes

## Abstract

**Aim:**

To explore the influence of the serum total bilirubin to total cholesterol (TBIL/TC) ratio on the progression of chronic kidney disease (CKD) in people with type 2 diabetes.

**Materials and Methods:**

The present retrospective discovery cohort investigated 4282 patients. The exposure was baseline TBIL/TC ratio. The outcome was the first time to progressing CKD, defined by a drop in the estimated glomerular filtration rate (eGFR) category, along with a reduction in eGFR of at least 25% compared to the baseline value. Hazard ratios (HRs) for CKD progression were evaluated based on the Cox proportional hazards approach. Dose–response relationships were conducted using Restricted Cubic Splines (RCS). Additionally, 758 patients were enrolled as an independent validation cohort.

**Results:**

During a median observation period of 2.4 years (interquartile range 1.3–3.8 years) within the discovery cohort, 522 individuals showed progression in CKD. The analysis revealed a negative association between the TBIL/TC ratio and the risk of CKD progression, with an adjusted HR of 0.17 and a 95% CI ranging from 0.07 to 0.41. After adjusting for confounding variables, the HRs for the second, third, and fourth quartiles of the TBIL/TC ratio were recorded at 0.61 (95% CI 0.48, 0.78), 0.55 (95% CI 0.42, 0.72), and 0.55 (95% CI 0.41, 0.74), respectively. Analysis with RCS indicated an optimal TBIL/TC ratio threshold of 0.25%. Similar results were also observed in the validation cohort.

**Conclusions:**

A higher TBIL/TC ratio was significantly associated with a reduced risk of CKD progression in patients with type 2 diabetes.

## Graphical abstract

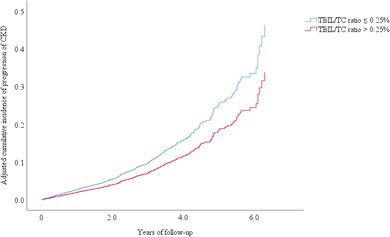

**Summary**

- The time‐based correlation between the bilirubin‐lipid ratio and chronic kidney disease in people with diabetes has not been well elaborated.
- This retrospective cohort study reported that a high level of serum total bilirubin to total cholesterol ratio was significantly associated with a low risk for chronic kidney disease progression in patients with type 2 diabetes. This suggests that it may be a novel prognostic factor in these populations.

## 1 Introduction

Chronic kidney disease (CKD) impacts between 8% and 16% of people worldwide [1], with around 130 million instances linked to type 2 diabetes mellitus. CKD due to diabetes accounted for 30.7% [2], and these conditions are major factors leading to end‐stage renal disease (ESKD) across many nations [3, 4]. Thus, it urgently needs to halt the progression of diabetes‐related kidney disease and search for biomarkers or easily accessible factors that can predict renal outcomes.

Recently, bilirubin has been regarded as a powerful endogenous antioxidant molecule [5] and more studies have indicated that bilirubin played a key role in the protective effect on diabetes. Further evidence indicates that bilirubin may offer protection owing to its ability to reduce inflammation, its cell‐shielding capabilities, and its properties against lipid peroxidation [6]. More importantly, several studies have shown an inverse correlation between serum bilirubin levels and diabetic kidney disease [7, 8], suggesting that serum bilirubin levels are excellent indicators for predicting the renal outcomes [9]. However, most of these studies were cross‐sectional studies or cohort studies with short follow‐up.

On the other hand, dyslipidemia is tightly related to the progression of CKD, especially in diabetes mellitus. Recent studies have reported that total cholesterol was a strong risk factor and had a positive relationship with progressive renal decline [10, 11]. Several epidemiological studies have also proposed that bilirubin, characterized by high lipophilicity, could be a novel indicator of coronary artery disease when combined with lipids; however, this hypothesis requires further validation across diverse populations [12, 13]. These researches provide new insight into the value of the combination of bilirubin and lipids.

To date, it is not exactly known the prognostic value of the bilirubin‐lipid ratio in patients with diabetes. Furthermore, no study has focused on the synergistic effect of the two indicators and evaluated the time‐based correlation between the bilirubin‐lipid ratio and CKD. Thus, in order to provide a reference and basis for first‐line clinical screening and reduce the risk for adverse renal outcomes, this study aims to investigate the relationship between the baseline ratio of total bilirubin to total cholesterol (TBIL/TC) and the subsequent development of CKD in patients with type 2 diabetes and then the effects of bilirubin on serum lipid levels and renal function.

## 2 Materials and Methods

### 2.1 Study Design and Participants

#### 2.1.1 Discovery Cohort

**FIGURE 1:**
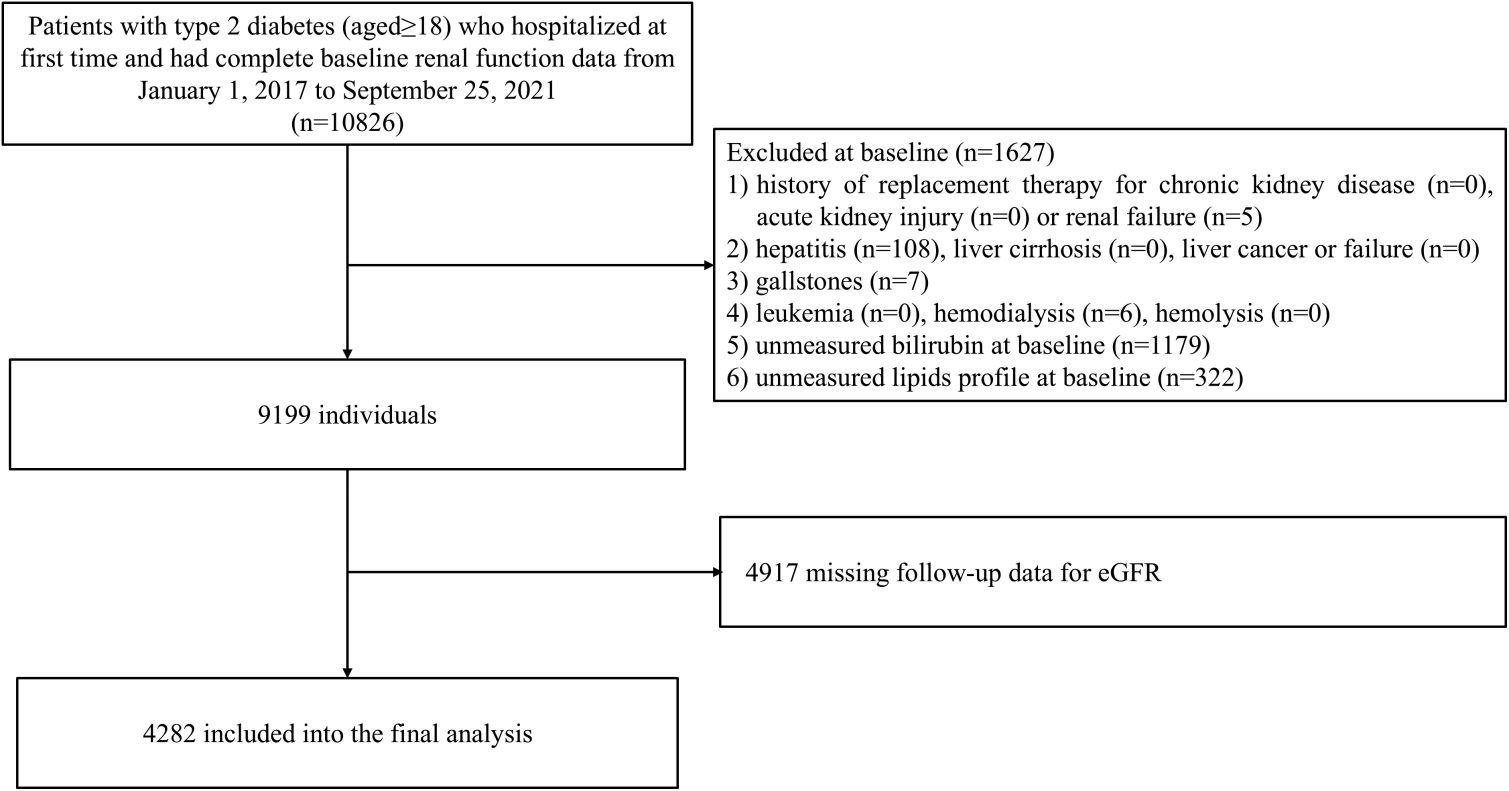
Flow chart of the discovery cohort.

This cohort selected patients with type 2 diabetes who visited Tianjin Medical University Chu Hsien‐I Memorial Hospital from January 1, 2017, to September 25, 2021, as subjects. Then, we included individuals who had complete data of spot urinary albumin‐creatinine ratio (UACR) and estimated glomerular filtration rate (eGFR) measurements approximately 5 years apart within the time window, aged ≥ 18 years and without a history of CKD replacement therapy, acute renal injury, or renal failure at the time of their first hospitalization. In addition, some related conditions that may have an impact on the exposure and outcomes were also excluded (i.e., hepatitis, liver cirrhosis, liver cancer or failure, gallstones, hemolysis, leukemia, hemodialysis). Based on the 2012 KDIGO guidelines [14], the diagnosis of acute renal injury was a comprehensive assessment by the clinician according to the patient's creatinine changes during the hospital stay. Since some patients may have multiple creatinine levels during the hospital stay and to more accurately assess baseline eGFR level, the first reported values were considered. Initially, a total of 10 826 patients were identified. Then, according to clinical diagnosis and further evaluation, 5 patients with renal failure who had not achieved renal replacement therapy and were not in the hemodialysis stage were excluded. 6 patients on hemodialysis were also excluded. Thus, 1627 patients were excluded, who did not meet the inclusion criteria or were missing baseline bilirubin and lipid profiles. Additionally, 4917 patients without follow‐up eGFR data were excluded. Ultimately, 4282 individuals with eGFR ≥ 15 mL/min/1.73 m^2^ were considered eligible. The local ethics committee of the institution approved the study protocol (approval number ZXYJNYYkMEC2023‐50). Figure 1 depicts the patient selection process for the discovery cohort.

#### 2.1.2 Validation Cohort

Furthermore, we chose as the validation cohort patients with type 2 diabetes who were initially hospitalized in the same institution from September 26, 2021, to June 25, 2022. The criteria for selecting and omitting participants aligned with the above discovery cohort. Ultimately, 758 individuals with complete data were incorporated into this analysis. Figure S1 depicts the patient selection process for the validation cohort.

### 2.2 Data Collection

At registration, participants reported their health background, received physical assessments, and underwent diagnostic testing. We collected demographic data, including medications for dyslipidemia, diabetes mellitus, and hypertension, tobacco and alcohol consumption habits, duration of diabetes, body mass index (BMI), age, gender, and systolic and diastolic blood pressure from medical records on the day of registration. Smoking and alcohol consumption statuses were self‐reported and categorized as either yes (former/current) or no (never). History of cardiovascular disease (CVD) was captured by self‐reported medical record or hospital diagnosis. Hypertension was defined by the use of antihypertensive medications or blood pressure ≥ 140/90 mmHg. Following a 12‐h fast during the night, blood specimens were taken and processed within a day with the help of an automated analyzer for clinical chemistry (Bayer, Germany). Laboratory tests included a complete blood profile, hemoglobin A_1c_ (HbA_1c_), tests for examining liver function (including total bilirubin, direct bilirubin, indirect bilirubin et al.), fasting lipid profile (including LDL‐cholesterol, HDL‐cholesterol, total cholesterol and triglycerides), creatinine, and uric acid. Glycemic control was determined by HbA_1c_ level (≤ 7% or > 7%). The UACR was measured using a spot urine sample. The eGFR was calculated using the Chronic Kidney Disease Epidemiology Collaboration (CKD‐EPI) formula [15]. eGFR values were categorized according to the stages outlined in the Kidney Disease: Improving Global Outcomes (KDIGO) guidelines: [16, 17] G1 ≥ 90, G2 60–89, G3a 45–59, G3b 30–44, G4 15–29, and G5 < 15 mL/min/1.73 m^2^. Converted baseline bilirubin units to mmol/L, divided the total bilirubin value by the total cholesterol value, and multiplied by 100 for calculating the TBIL/TC ratio (%) [13]. Then categorized this ratio into quartiles: Q1 (≤ 0.18%), Q2 (0.18%–0.25%), Q3 (0.25%–0.34%), and Q4 (> 0.34%).

### 2.3 Exposure, Outcome, and Follow‐Up Periods

#### 2.3.1 Exposure

The exposure was baseline TBIL/TC ratio, examined as both a continuous variable and quartile categories.

#### 2.3.2 Outcome

The outcome was the first time progressing CKD, characterized by a decline in the category of eGFR along with a reduction of 25% or more from the baseline eGFR values [18, 19]. The study follow‐up ended on September 25, 2023. For each participant, the final observation date was either the date when the study outcome occurred or the date of the last recorded eGFR prior to the set cutoff date if the outcome did not occur [20].

### 2.4 Statistical Analysis

Baseline characteristics were displayed using average values with standard deviation, median values with IQR, or proportions. Missing values, which were less than 0.9% for HbA_1c_, BMI, and duration of diabetes, were addressed through multiple imputation. We compared differences in means, proportions, or medians of baseline data across the TBIL/TC ratio quartile groups using ANOVA, the *χ* ^2^ test, or the Kruskal‐Wallis test, as appropriate. When dividing patients into two groups, the Mann–Whitney *U* test, *t*‐test, or *χ* ^2^ test were employed for assessing differences of groups. Cox proportional hazards regression models examined the correlations of the TBIL/TC ratio with eGFR category progression, considering the ratio as both a continuous variable and a categorical variable in quartiles. The adjustment variables included in the regression analyzes were selected according to the following criteria: (1) demographic and clinical data and reported in the relevant literature [8, 20], and (2) variables with *p* < 0.05 in univariate analysis, including age, systolic blood pressure, glycemic control, insulin treatment (yes or no), use of statin (yes or no), use of ACE inhibitors or angiotensin receptor blockers (ACEi/ARBs) (yes or no), logarithmically transformed triglycerides, duration of diabetes, LDL‐cholesterol, ALT and logarithmically transformed UACR. Three different models were performed, including a crude model only considering the TBIL/TC ratio, the second model adjusted for age and sex, and the third multivariable model adjusted for all confounders mentioned above. Collinearity analysis demonstrated no collinearity among the variables (variance inflation factor < 2). Schoenfeld residuals were carried out for verifying the proportional hazards assumption before this analysis. Kaplan–Meier curves were employed for calculating the cumulative incidence rates of CKD progression.

Additionally, we explored the dose–response link of the TBIL/TC ratio and the outcome by using a multivariable‐adjusted restricted cubic spline (RCS) curve with four knots, which can take into account the smoothness of the curve and avoid the reduction of accuracy caused by overfitting [21]. Then, we categorized the baseline TBIL/TC ratio based on the optimal threshold value derived from the spline model. The median value served as the reference (hazard ratio = 1). The subgroup analysis was further stratified by gender, age (< 65 years or ≥ 65 years), BMI (≤ 24 kg/m^2^ or > 24 kg/m^2^), duration of diabetes (≤ 10 years or > 10 years), and HbA_1c_ (≤ 7% or > 7%).

Several sensitivity analyzes were also conducted. First, subjects were stratified by proteinuria category and CKD status to elaborate associations between TBIL/TC ratio and study outcome in subgroups. Second, we further adjusted baseline eGFR. Third, the multivariate model further adjusted for SGLT2i and GLP‐1RA use. Fourth, smoking status (*n* = 3743) and alcohol consumption status (*n* = 3674) were added as covariates in the multivariable model for participants with available data. Fifth, taking into account the potential confounding effects of CVD and hypertension, they were both further incorporated into the regression model for adjustment (*n* = 3024). Sixth, further analysis was performed in patients with no SGLT2i and GLP‐1RA use at baseline (*n* = 3208). Seventh, we additionally adjusted baseline uric acid in the multivariable model, which is also known to be associated with renal outcomes [22]. And finally, we performed RCS analyzes with different knot placements to ensure robustness such as 3‐knot and 5‐knot in the RCS regression. All statistical analyzes were carried out by SPSS version 26 and R version 4.2.3 software. A two‐tailed *p*‐value < 0.05 means statistical significance.

## 3 Results

### 3.1 Baseline Characteristics

The clinical and laboratory data of the discovery cohort according to TBIL/TC ratio quartiles are listed in Table 1. Among the 4282 eligible individuals, there were 2366 (55.3%) male and 1916 (44.7%) female with an average age of 56.3 ± 12.1 years. The mean baseline eGFR reached 97.96 ± 21.12 mL/min/1.73 m^2^. No patients had serum bilirubin levels in the toxic range. As the quartiles increase, patients with higher TBIL/TC ratios have lower ages, lower baseline UACR and higher baseline eGFR levels, lower HbA_1c_ levels, and shorter diabetes durations. Additionally, the use of medications was less frequent in patients in the highest quartile when contrasted with those in the lowest quartile.

**TABLE 1:** Baseline clinical characteristics of participants based on TBIL/TC ratio quartiles (discovery cohort).

Variable	Total (*n* = 4282)	Q1 (≤ 0.18%) (*n* = 1106)	Q2 (0.18%–0.25%) (*n* = 1097)	Q3 (0.25%–0.34%) (*n* = 1050)	Q4 (> 0.34%) (*n* = 1029)	*p*
Male, *n* (%)	2366 (55.3)	426 (38.5)	546 (49.8)	654 (62.3)	740 (71.9)	< 0.001
Age, years	56.3 ± 12.1	56.8 ± 11.7	56.6 ± 12.2	56.2 ± 11.9	55.4 ± 12.6	0.045
Diabetic duration, years	10.33 ± 7.71	11.13 ± 7.97	10.26 ± 7.65	10.17 ± 7.54	9.67 ± 7.58	< 0.001
Smoking, *n* (%)^a^	1483 (39.6)	314 (33.1)	352 (36.9)	407 (44.3)	410 (44.5)	< 0.001
Alcohol consumption, *n* (%)^b^	1372 (37.3)	247 (26.6)	321 (34.4)	390 (43.0)	414 (45.6)	< 0.001
History of CVD, *n* (%)^c^	753 (24.9)	211 (26.9)	177 (23.0)	180 (24.4)	185 (25.2)	0.345
Hypertension, *n* (%)^d^	1713 (56.6)	475 (60.7)	416 (54.2)	409 (55.3)	413 (56.3)	0.054
BMI, kg/m^2^	27.18 ± 10.74	26.63 ± 3.89	27.86 ± 16.56	27.25 ± 11.91	26.97 ± 5.08	0.052
Diastolic BP, mmHg	80.90 ± 10.62	80.49 ± 10.77	80.64 ± 10.43	81.38 ± 11.08	81.11 ± 10.13	0.185
Systolic BP, mmHg	135.01 ± 18.07	137.15 ± 19.25	134.91 ± 17.31	134.64 ± 18.22	133.21 ± 17.16	< 0.001
HbA_1c_, %	8.76 ± 1.97	8.94 ± 2.10	8.80 ± 1.98	8.69 ± 1.90	8.59 ± 1.87	< 0.001
HbA_1c_ > 7%, *n* (%)	3405 (79.5)	902 (81.6)	881 (80.3)	815 (77.6)	807 (78.4)	0.097
Total cholesterol, mmol/L	5.09 ± 1.36	6.10 ± 1.55	5.22 ± 1.02	4.77 ± 0.97	4.17 ± 0.98	< 0.001
HDL‐cholesterol, mmol/L	1.12 ± 0.28	1.20 ± 0.31	1.14 ± 0.26	1.09 ± 0.26	1.04 ± 0.25	< 0.001
Triglyceride, mmol/L	1.65 (1.16, 2.42)	1.96 (1.41, 3.00)	1.68 (1.19, 2.46)	1.58 (1.12, 2.22)	1.39 (1.00, 2.01)	< 0.001
LDL‐cholesterol, mmol/L	3.37 ± 1.00	4.08 ± 1.06	3.47 ± 0.82	3.16 ± 0.77	2.71 ± 0.78	< 0.001
Uric acid, umol/L	331.06 ± 93.76	335.50 ± 98.56	326.66 ± 89.94	332.41 ± 92.25	329.62 ± 93.76	0.147
Indirect bilirubin, umol/L	9.87 ± 4.47	6.13 ± 2.05	8.51 ± 2.26	10.51 ± 2.68	14.71 ± 4.98	< 0.001
Direct bilirubin, umol/L	3.44 ± 1.79	2.28 ± 1.00	2.88 ± 1.00	3.53 ± 1.23	5.18 ± 2.21	< 0.001
Total bilirubin, umol/L	13.31 ± 5.56	8.40 ± 2.11	11.38 ± 2.30	14.04 ± 2.92	19.89 ± 5.97	< 0.001
TBIL/TC ratio, %	0.28 ± 0.15	0.14 ± 0.03	0.22 ± 0.02	0.30 ± 0.03	0.49 ± 0.16	< 0.001
GGT, U/L	36.16 ± 37.84	33.00 ± 29.74	36.53 ± 41.13	36.56 ± 36.01	38.73 ± 43.17	0.005
AST, U/L	22.44 ± 14.30	20.63 ± 13.90	21.98 ± 13.34	22.75 ± 13.44	24.54 ± 16.17	< 0.001
ALT, U/L	25.23 ± 21.81	21.54 ± 19.59	24.47 ± 20.66	26.34 ± 20.97	28.88 ± 25.16	< 0.001
Baseline UACR, mg/g	10.77 (5.92, 36.16)	14.72 (6.94, 83.47)	10.54 (5.84, 31.81)	10.11 (5.75, 35.42)	9.12 (5.22, 22.33)	< 0.001
Baseline eGFR, mL/min/1.73 m^2^	97.96 ± 21.12	91.13 ± 25.25	96.45 ± 20.67	97.30 ± 18.79	99.25 ± 17.81	< 0.001
Statins use, *n* (%)	2577 (60.2)	797 (72.1)	687 (62.6)	600 (57.1)	493 (47.9)	< 0.001
ACEi/ARBs use, *n* (%)	1569 (36.6)	434 (39.2)	360 (32.8)	420 (40.0)	355 (34.5)	< 0.001
Insuline use, *n* (%)	2496 (58.3)	697 (63.0)	644 (58.7)	587 (55.9)	568 (55.2)	< 0.001
SGLT2i use, *n* (%)	769 (18.0)	242 (21.9)	181 (16.5)	163 (15.5)	183 (17.8)	< 0.001
GLP‐1RA use, *n* (%)	438 (10.2)	104 (9.4)	111 (10.1)	122 (11.6)	101 (9.8)	0.357

*Note:* Data are expressed as mean ± SD, median (IQR), or *n* (%).

Abbreviations: ACEi, angiotensin‐converting enzyme inhibitor; ALT, alanine aminotransferase; ARBs, angiotensin II receptor blockers; AST, aspertate aminotransferase; BMI, body mass index; BP, blood pressure; CVD, cardiovascular disease; eGFR, estimated glomerular filtration rate; GGT, gamma‐glutamyl transpeptidase; GLP‐1RA, glucagon‐like peptide 1 receptor agonists; HbA_1c_, hemoglobin A_1c_; SGLT2i, sodium‐glucose cotransporter 2 inhibitors; TBIL/TC ratio, serum total bilirubin to total cholesterol ratio; UACR, urinary albumin‐creatinine ratio.

^a^ Participant with available smoking data at baseline (former/current or never) (*n* = 3743).

^b^ Participants with available alcohol consumption status data at baseline (former/current or never) (*n* = 3674).

^c^ Participants with a history of CVD at baseline (*n* = 3024).

^d^ Participants with hypertension at baseline (*n* = 3024).

Out of all participants, 522 (12.2%) experienced the outcome over an average follow‐up duration of 2.4 years, with an IQR from 1.3 to 3.8 years. During this period, the median frequency of eGFR measurements per participant was 1.3 annually, with an IQR of 0.7–2.2. Participants with CKD progression had a lower baseline TBIL/TC ratio in comparison to those without events. Characteristics of subjects stratified by the outcome were shown in Table S1.

### 3.2 Association of Baseline TBIL/TC Ratio With CKD Progression in Discovery Cohort

Univariate Cox regression analysis showed that TBIL/TC ratio was significantly associated with CKD progression (Table S3). Then, we employed three Cox regression models for exploring the independent correlation of the baseline TBIL/TC ratio and the outcome. The TBIL/TC ratio was inversely linked to the outcome in both the unadjusted model (HR 0.03, 95% confidence interval [CI] 0.01, 0.07) and the fully adjusted model (HR 0.17, 95% CI 0.07, 0.41). When treated as a categorical variable, participants with TBIL/TC ratios in quartiles 2–4 had lower risks of CKD progression (unadjusted HR 0.46 [95% CI 0.37, 0.57], 0.40 [95% CI 0.32, 0.51], and 0.37 [95% CI 0.29, 0.47], separately) in comparison to those in quartile 1. The correlation remained significant after adjusting for sex and age in model 2 and further adjusting for possible confounders in model 3 (adjusted HR 0.61 [95% CI 0.48, 0.78], 0.55 [95% CI 0.42, 0.72], and 0.55 [95% CI 0.41, 0.74], separately) (Table 2).

**TABLE 2:** HRs (95% CIs) for progression of CKD based on baseline TBIL/TC ratio (discovery cohort).

TBIL/TC ratio	Events/total	Model 1	Model 2	Model 3
		HR (95% CI) *p*	HR (95% CI) *p*	HR (95% CI) *p*
Continuous (per unit)	522/4282 (12. 2)	0.03 (0.01, 0.07) < 0.001	0.02 (0.01, 0.05) < 0.001	0.17 (0.07, 0.41) < 0.001
Quartiles (%)				
Q1 (≤ 0.18)	220/1106 (19.9)	Reference	Reference	Reference
Q2 (0.18–0.25)	117/1097 (10.7)	0.46 (0.37, 0.57) < 0.001	0.43 (0.34, 0.54) < 0.001	0.61 (0.48, 0.78) < 0.001
Q3 (0.25–0.34)	99/1050 (9.4)	0.40 (0.32, 0.51) < 0.001	0.36 (0.28, 0.46) < 0.001	0.55 (0.42, 0.72) < 0.001
Q4 (> 0.34)	86/1029 (8.4)	0.37 (0.29, 0.47) < 0.001	0.32 (0.25, 0.41) < 0.001	0.55 (0.41, 0.74) < 0.001

*Note:* Model 1 crude. Model 2 adjusted by sex and age. Model 3 adjusted for variables with *p* value < 0.05 in univariate analysis plus sex and BMI, including sex, age, BMI, systolic blood pressure, glycemic control, insulin treatment (yes or no), use of statin (yes or no), use of ACE inhibitors or angiotensin receptor blockers (ACEi/ARBs) (yes or no), logarithmically transformed triglycerides, duration of diabetes, LDL‐cholesterol, ALT and logarithmically transformed UACR.

**FIGURE 2:**
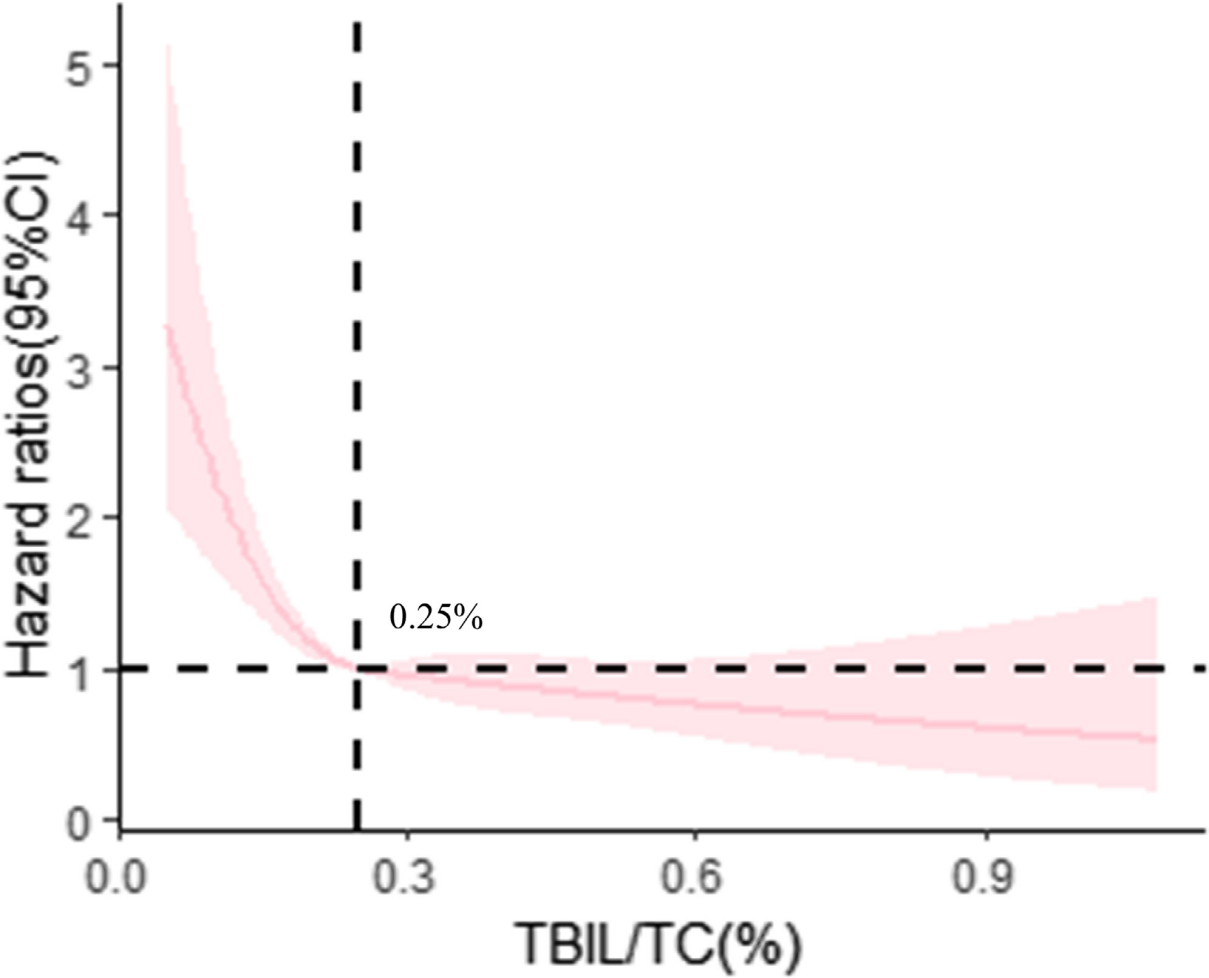
The association between the TBIL/TC ratio and CKD progression (discovery cohort). The following covariates at baseline were incorporated into the restricted cubic spline model with four knots (5th, 35th, 65th, and 95th percentile levels of TBIL/TC ratio): Sex, age, BMI, systolic blood pressure, glycemic control, insulin treatment (yes or no), use of statin (yes or no), use of ACE inhibitors or angiotensin receptor blockers (ACEi/ARBs) (yes or no), logarithmically transformed triglycerides, duration of diabetes, LDL‐cholesterol, ALT, and logarithmically transformed UACR.

**FIGURE 3:**
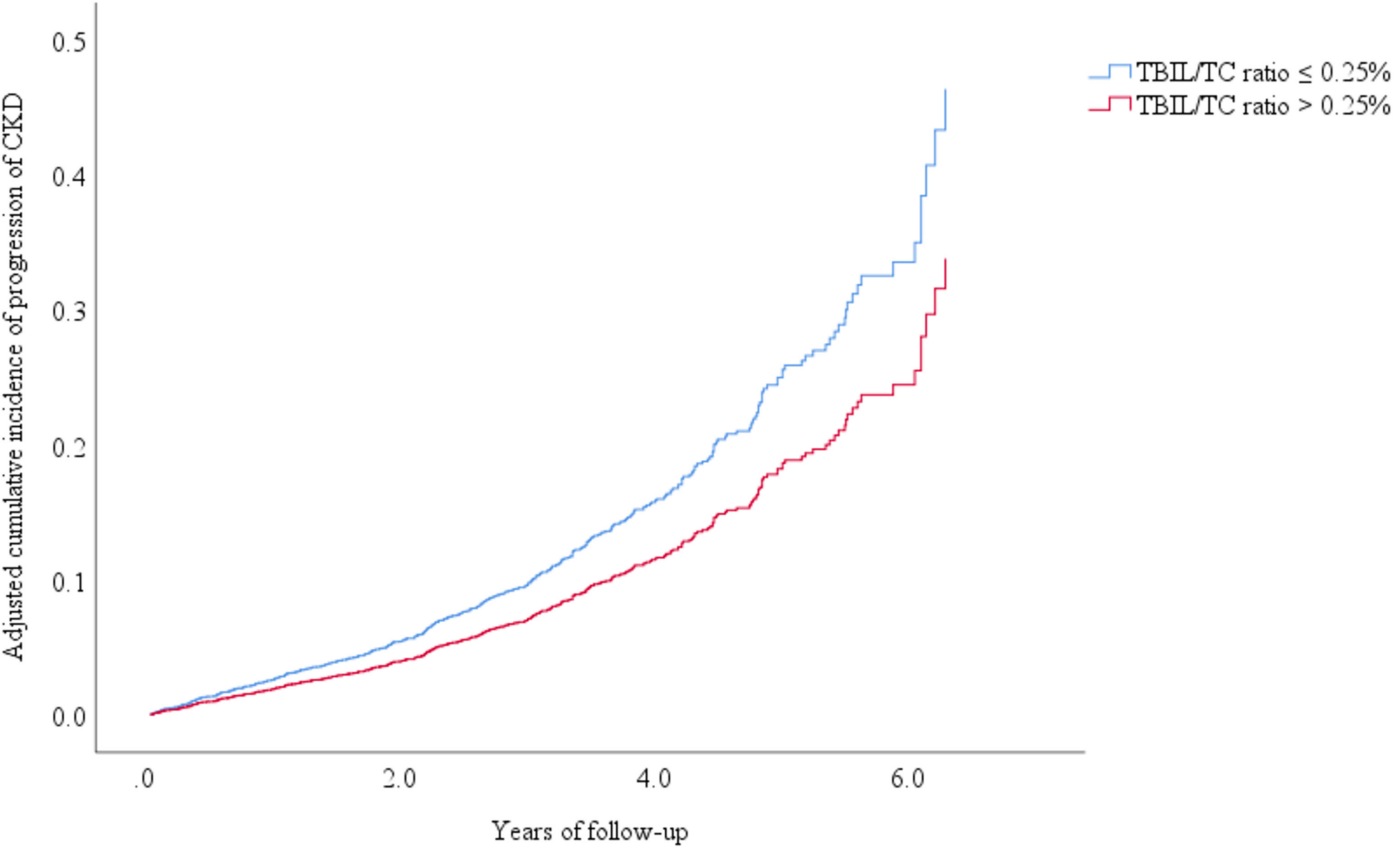
Cumulative incidence of CKD progression stratifying by threshold value of the TBIL/TC ratio (discovery cohort). The following covariates at baseline were incorporated into the model: Sex, age, BMI, systolic blood pressure, glycemic control, insulin treatment (yes or no), use of statin (yes or no), use of ACE inhibitors or angiotensin receptor blockers (ACEi/ARBs) (yes or no), logarithmically transformed triglycerides, duration of diabetes, LDL‐cholesterol, ALT, and logarithmically transformed UACR.

Subsequently, a multivariable‐adjusted RCS model was fitted to show the dose–response correlation of baseline TBIL/TC ratio and CKD progression in Figure 2. The optimal TBIL/TC ratio threshold value was 0.25%. Then, participants were divided into two groups based on the TBIL/TC ratio threshold (low TBIL/TC ratio ≤ 0.25% and high TBIL/TC ratio > 0.25%) and their baseline characteristics were analyzed (Table S2). Individuals with a high TBIL/TC ratio had lower risks of CKD progression than those with a low TBIL/TC ratio (adjusted HR 0.73 [95% CI 0.59, 0.90]), as indicated by multivariate Cox regression analysis (Table 3). In addition, the adjusted cumulative incidence of CKD progression grouped by TBIL/TC ratio reference point was shown in Figure 3.

**TABLE 3:** Threshold effect analysis of TBIL/TC ratio on progression of CKD (discovery cohort).

	Events/total	Model 1		Model 2		Model 3	
		HR (95% CI)	*p*	HR (95% CI)	*p*	HR (95% CI)	*p*
Low TBIL/TC ratio (≤ 0.25%)	337/2203 (15.3)	Reference	—	Reference	—	Reference	—
High TBIL/TC ratio (> 0.25%)	185/2079 (8.9)	0.54 (0.45, 0.65)	< 0.001	0.51 (0.42, 0.61)	< 0.001	0.73 (0.59, 0.90)	0.003

*Note:* Model 1 crude. Model 2 adjusted by sex and age. Model 3 adjusted for variables with *p* value < 0.05 in univariate analysis plus sex and BMI, including sex, age, BMI, systolic blood pressure, glycemic control, insulin treatment (yes or no), use of statin (yes or no), use of ACE inhibitors or angiotensin receptor blockers (ACEi/ARBs) (yes or no), logarithmically transformed triglycerides, duration of diabetes, LDL‐cholesterol, ALT and logarithmically transformed UACR.

**FIGURE 4:**
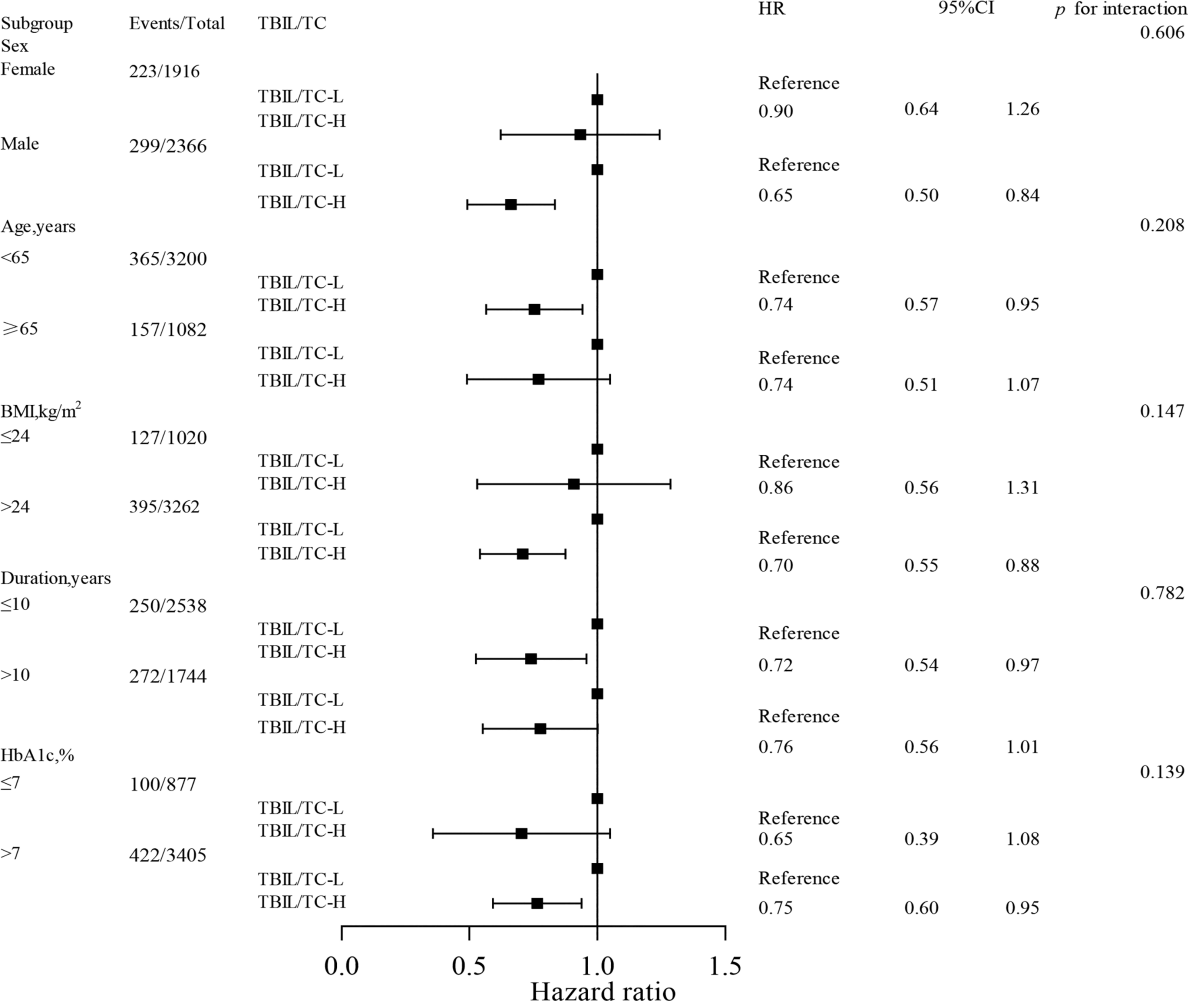
Subgroup analysis of the association between TBIL/TC ratio and CKD progression (discovery cohort). TBIL/TC‐L means low TBIL/TC ratio (≤ 0.25%); TBIL/TC‐H means high TBIL/TC ratio (> 0.25%); Duration means duration of diabetes. The following covariates at baseline were incorporated into the model (except stratification variables): Sex, age, BMI, systolic blood pressure, glycemic control, insulin treatment (yes or no), use of statin (yes or no), use of ACE inhibitors or angiotensin receptor blockers (ACEi/ARBs) (yes or no), logarithmically transformed triglycerides, duration of diabetes, LDL‐cholesterol, ALT, and logarithmically transformed UACR.

In the subgroup analysis, high TBIL/TC ratio versus low TBIL/TC ratio revealed a consistent association with a reduced risk of CKD progression across various subgroups stratified by gender, HbA_1c_, duration of diabetes, BMI, and age (Figure 4). More importantly, among male patients, patients aged < 65 years, patients with high BMI > 24 kg/m^2^ and poor glycemic control, the predictive value of TBIL/TC ratio was more pronounced (all interaction *p*‐values > 0.05).

### 3.3 Sensitivity Analyzes

The findings from sensitivity analyzes were consistent with those from the primary analyzes. Specifically, regarding patients with a low TBIL/TC ratio as the reference in the multivariable Cox regression, similar findings are shown in the following: (1) The link between the TBIL/TC ratio and CKD progression risk was generally consistent across albuminuria categories (30–300 mg/g or ≥ 300 mg/g) and CKD status (eGFR ≤ 60 or > 60 mL/min/1.73 m^2^) (Tables S4 and S5). (2) Similar outcomes were found when additionally adjusted for baseline eGFR or SGLT2i and GLP‐1RA use (Table S6). (3) The results remained consistent when smoking status and alcohol consumption status at baseline were included as additional covariates in the multivariable models (Table S6). (4) The results remained essentially unchanged when taking into account the potential confounding effects of CVD and hypertension (Table S6). (5) Consistent results were also seen in patients with no SGLT2i and GLP‐1RA use at baseline (Table S6). (6) Similar results were still obtained after additional adjustments for uric acid levels (Table S6). Finally, different knot placements in the RCS model showed similar trends compared with the primary analyzes findings (Figure S2).

### 3.4 Association of Baseline TBIL/TC Ratio With CKD Progression in Validation Cohort

In the 758 validation cohort participants, 65 (8.6%) study outcomes were identified over a median follow‐up period of 1.1 years, with an IQR of 0.7 to 1.4 years. In addition, no significant differences in sex, BMI, age, HbA_1c_, systolic blood pressure, and baseline UACR were found between the discovery cohort and the validation cohort (Table S7). When the same model analyzes were performed in the validation cohort, a significant correlation between baseline TBIL/TC ratio and CKD progression was still observed (Table S8).

## 4 Discussion

In this single‐center, retrospective discovery cohort study, the results indicated that the baseline TBIL/TC ratio was significantly associated with CKD progression, irrespective of recognized risk factors like baseline albuminuria and eGFR. Further analysis using RCS plots indicated that a lower TBIL/TC ratio might be effective in identifying those with an increased risk of CKD progression. Furthermore, an independent validation cohort and several sensitivity analyzes have enhanced the robustness of these observations.

At present, approximately 132.3 million people have CKD in China, with 31.08 million cases attributable to type 2 diabetes [2]. The combination of diabetes and CKD has emerged as the foremost cause of hospitalization for CKD [23]. It is worth noting that diabetic kidney disease, which is manifested by continuous albumin presence in the urine and/or a steady decrease in glomerular filtration rate, affects 25%–40% of diabetic patients [24] and emerges as the primary reason for ESKD globally. However, there is often a delay in the diagnosis of diabetic kidney disease, particularly in the initial stages when the reduction in eGFR is minimal or albuminuria is low. Therefore, early evaluation is urgent for managing risk and delaying the advancement of diabetes‐related kidney disease.

Two central mechanisms implicated in the development and advancement of diabetic kidney disease are the metabolic pathway and the inflammatory pathway [25]. Lipids are known to contribute to the deterioration of renal function (eGFR) through lipotoxicity, which activates cell death, mitochondrial dysfunction, oxidative stress, and inflammation [26, 27]. Notably, emerging evidence suggests that bilirubin is considered an ideal antioxidant against lipid peroxidation [6, 28] due to its anti‐inflammatory [29], antioxidant [30], and lipid‐modulating properties. One study suggested that serum bilirubin, when combined with various lipids, could improve the prediction of coronary artery disease more effectively than traditional blood lipids alone, though further validation in other populations was needed [12]. In the current study, we found better lipid profiles in patients with high bilirubin levels, which was in agreement with previous studies on the relationship between serum bilirubin and the lipoprotein spectrum [31]. More importantly, we revealed that patients with a high TBIL/TC ratio had a lower risk of CKD progression, suggesting that the TBIL/TC ratio could be a reliable prognostic indicator and may predict poor renal outcomes in individuals with type 2 diabetes.

So far, no research has examined the relationship between the TBIL/TC ratio and CKD. Existing studies primarily investigated the association of simple serum bilirubin and kidney health outcomes. For instance, a decade‐long observational cohort study in Japan involving diabetic patients showed that initial total bilirubin levels were inversely associated with kidney function decline and independently predicted a decrease in eGFR [9]. Cross‐sectional studies of individuals with type 2 diabetes have also found positive associations between eGFR and serum bilirubin levels, and inverse relationships with urinary albumin levels [32]. Nevertheless, the research presents inconsistencies, as some studies report no link between serum bilirubin levels and diabetic kidney disease [7], and others suggest that high bilirubin levels might even pose a risk [33]. Differences in lifestyle and genetic factors among various populations may contribute to these inconsistent conclusions.

Identifying special type 2 diabetic patients at high risk of renal disease progression has important public health implications. Here, we observed that the predictive value of a higher TBIL/TC ratio was more pronounced in male patients aged < 65 years with a high BMI > 24 kg/m^2^ and poor glycemic control by subgroup analyzes, indicating that these individuals may face more severe kidney‐related events. Similarly, it has been widely reported that variations in sex and age affect the relationship between renal outcomes and serum bilirubin levels, as cited in references [34, 35] a trend that was also observed in our study.

At present, despite existing certain types of medications such as SGLT2i and GLP‐1RA therapies being available for patients with T2D and CKD, epidemiological data suggest that the residual risk of renal failure remains. This highlights the need to adopt more effective biomarkers for disease management to identify high‐risk populations in clinical practice. In the current study, to further rule out the influence of SGLT2i or GLP‐1RA treatment on the renal outcomes in diabetes, data were reanalyzed after excluding patients taking SGLT2i or GLP‐1RA drugs. The results did not change significantly. Similar results were also observed after additional adjustments for certain types of medications in those patients in sensitivity analysis and indicating that the robustness of the main analysis results was applicable to almost all populations. Conclusively, our results contribute new insights and support to the practice of integrating bilirubin and lipid measurements in diabetic patients.

Various potential mechanisms may account for the link of TBIL/TC ratio and kidney disease progression. Preclinical studies have demonstrated that bilirubin acts to suppress triglycerides and cholesterol in the serum and that bilirubin's safeguarding impact on the renal function of diabetic rats is related not only to the scavenging of reactive oxygen species [36] but also to the alleviation of renal dyslipidemia [37]. One theory proposes that bilirubin serves as a natural antioxidant, impeding the oxidation of LDL and the development of foam cells [38], while bilirubin can also solubilize and excrete total cholesterol, which leads to reduced LDL‐cholesterol levels and increased HDL‐cholesterol levels [39]. Furthermore, in vitro experimental studies exploring bilirubin deficiency have shown signs of hyperlipidemia, endothelial dysfunction, and increased systemic oxidative stress [40]. Additionally, it is widely reported that serum total bilirubin levels are inversely related to the development and progression of diabetic nephropathy [32, 41]. Above all, those data imply that bilirubin may hinder kidney disease progression by inhibiting lipid oxidation and proinflammatory responses, further attenuating renal lipotoxicity and vascular endothelial activation [42].

Our findings highlight the importance of the TBIL/TC ratio as a useful marker for assessing the risk of CKD advancement in patients with type 2 diabetes. The ratio offers critical insights into the health status of patients with both type 2 diabetes and CKD, serving as a straightforward but powerful tool for predicting disease trajectory. Further research and confirmatory studies are also required to validate these results and delve into the underlying processes by which the TBIL/TC ratio may influence kidney function in individuals with diabetes.

However, this research also has its limitations. First, inherent to the retrospective cohort design and the relatively short follow‐up time, there was a possibility of underestimating CKD progression. Second, employing only one eGFR reading at the outset and during subsequent follow‐up could have resulted in incorrect categorization of eGFR levels, although the study minimized this error by using eGFR stage progression with an endpoint defined as a ≥ 25% decline from baseline. Third, the study did not evaluate variations over time in lab results, blood pressure measurements, BMI, and medication usage throughout the follow‐up period. Fourth, the investigation did not account for baseline hemoglobin levels, which may affect total bilirubin levels. Finally, the study population was from hospitalized patients instead of a stable outpatient pool and was drawn from a single ethnic group, constraining the broader applicability of the results.

In conclusion, this study suggests that an elevated TBIL/TC ratio correlates with a reduced risk of CKD progression. The TBIL/TC ratio provides a simple and robust new observational index and could be employed as a tool for risk stratification and monitoring the progression of CKD in patients with type 2 diabetes.

## Disclosure

The authors have nothing to report.

## Conflicts of Interest

The authors declare no conflicts of interest.

## Supporting information


**Data S1.** Supporting Information.
