# Supplementary material for: Association of Serum Total Bilirubin to Cholesterol Ratio With Progression of Chronic Kidney Disease in Patients With Type 2 Diabetes: A Retrospective Cohort Study
Source: J Diabetes. 2025 May 13;17(5):e70097. doi: 10.1111/1753-0407.70097 (PMC12069979; doi:10.1111/1753-0407.70097)
Supplement: Supplementary file 1 — Data S1. Supporting Information. [file JDB-17-e70097-s001.docx]

***Supplementary Material***

**Association of serum total bilirubin to cholesterol ratio with progression of chronic kidney disease in patients with type 2 diabetes: a retrospective cohort study**

Yanyan Chen ^1†^, Shanshan Wang ^1†^, Hang Guo ^1^, Fei Han ^1^, Bei Sun ^1^, Nan Li ^2^, Hongxi Yang ^3^, Liming Chen ^1*^

**^*^Correspondence:** Liming Chen; xfx22081@vip.163.com

Table S1 Baseline characteristics of participants stratifying by the outcome (discovery cohort)

|  | Progression of chronic kidney disease | |  |
| --- | --- | --- | --- |
| Variable | No (n=3760) | Yes (n=522) | *P* value |
| Male, n (%) | 2067 (55.0) | 299 (57.3) | 0.321 |
| Age, years | 55.9±12.2 | 59.0±10.9 | <0.001 |
| Diabetic duration, years | 10.00±7.56 | 12.67±8.34 | <0.001 |
| Smoking, n (%) ^a^ | 1297 (39.2) | 186 (43.2) | 0.111 |
| Alcohol consumption, n (%) ^b^ | 1220 (37.5) | 152 (35.8) | 0.499 |
| History of CVD, n (%) ^c^ | 652 (24.4) | 101 (29.1) | 0.054 |
| Hypertension, n (%) ^d^ | 1446 (54.0) | 267 (77.0) | <0.001 |
| BMI, kg/m^2^ | 27.17±10.71 | 27.24±10.95 | 0.882 |
| Diastolic BP, mmHg | 80.73±10.43 | 82.09±11.82 | 0.013 |
| Systolic BP, mmHg | 134.13±17.51 | 141.39±20.62 | <0.001 |
| HbA1c,% | 8.72±1.94 | 9.00±2.18 | 0.006 |
| HbA1c>7%, n (%) | 2983 (79.3) | 422 (80.8) | 0.424 |
| Total cholesterol, mmol/L | 5.05±1.31 | 5.37±1.62 | <0.001 |
| HDL-cholesterol, mmol/L | 1.12±0.27 | 1.14±0.31 | 0.056 |
| Triglyceride, mmol/L | 1.64 (1.15,2.38) | 1.77 (1.21,2.81) | <0.001 |
| LDL-cholesterol, mmol/L | 3.35±0.97 | 3.53±1.17 | <0.001 |
| Uric acid, umol/L | 326.60±92.18 | 363.21±98.67 | <0.001 |
| Indirect bilirubin, umol/L | 10.06±4.46 | 8.54±4.29 | <0.001 |
| Direct bilirubin, umol/L | 3.50±1.81 | 2.95±1.56 | <0.001 |
| Total bilirubin, umol/L | 13.56±5.55 | 11.49±5.23 | <0.001 |
| TBIL/TC ratio, % | 0.29±0.15 | 0.24±0.14 | <0.001 |
| GGT, U/L | 36.31±37.73 | 35.08±38.66 | 0.488 |
| AST, U/L | 22.61±13.78 | 21.19±17.59 | 0.033 |
| ALT, U/L | 25.77±21.37 | 21.38±24.38 | <0.001 |
| Baseline UACR, mg/g | 10.77 (5.92,36.16) | 49.00 (9.68,788.05) | <0.001 |
| Baseline eGFR, mL/min/1.73 m^2^ | 98.11±19.50 | 80.45±25.43 | <0.001 |
| Statins use, n (%) | 2230 (59.3) | 347 (66.5) | 0.002 |
| ACEi/ARBs use, n (%) | 1308 (34.8) | 261 (50.0) | <0.001 |
| Insuline use, n (%) | 2106 (56.0) | 390 (74.7) | <0.001 |
| SGLT2i use, n (%) | 669 (17.8) | 100 (19.2) | 0.447 |
| GLP-1RA use, n (%) | 398 (10.6) | 40 (7.7) | 0.039 |

Data are expressed as mean ± SD, median (IQR), or n (%).

Abbreviations: CVD, cardiovascular disease; BMI, body mass index; BP, blood pressure; HbA_1c_, hemoglobin A_1c_; TBIL/TC ratio, serum total bilirubin to total cholesterol ratio; GGT, gamma-glutamyl transpeptidase; AST, aspertate aminotransferase; ALT, alanine aminotransferase; UACR, urinary albumin-creatinine ratio; eGFR, estimated glomerular filtration rate; ACEi, angiotensin-converting enzyme inhibitor; ARBs, angiotensin II receptor blockers; SGLT2i, sodium-glucose cotransporter 2 inhibitors; GLP-1RA, glucagon-like peptide 1 receptor agonists.

^a^ participants with available smoking data at baseline (former/current or never) (n=3743)

^b^ participants with available alcohol consumption status data at baseline (former/current or never) (n=3674)

^c^ participants with history of CVD at baseline (n= 3024)

^d^ participants with hypertension at baseline (n= 3024)

Table S2 Baseline characteristics of participants stratifying by threshold value of the TBIL/TC ratio (discovery cohort)

| Variable | Low TBIL/TC ratio  (≤0.25%, n=2203) | High TBIL/TC ratio  (>0.25%, n=2079) | *P* value |
| --- | --- | --- | --- |
| Male, n (%) | 972 (44.1) | 1394 (67.1) | <0.001 |
| Age, years | 56.7±12.0 | 55.8±12.3 | 0.017 |
| Diabetic duration, years | 10.70±7.83 | 9.93±7.56 | 0.001 |
| Smoking, n (%) ^a^ | 666 (35.0) | 817 (44.4) | <0.001 |
| Alcohol consumption, n (%) ^b^ | 568 (30.5) | 804 (44.3) | <0.001 |
| History of CVD, n (%) ^c^ | 388 (25.0) | 365 (24.8) | 0.880 |
| Hypertension, n (%) ^d^ | 891(57.4) | 822 (55.8) | 0.362 |
| BMI, kg/m^2^ | 27.24±12.02 | 27.11±9.19 | 0.701 |
| Diastolic BP, mmHg | 80.57±10.60 | 81.25±10.63 | 0.036 |
| Systolic BP, mmHg | 136.03±18.34 | 133.93±17.72 | <0.001 |
| HbA_1c_,% | 8.87±2.04 | 8.64±1.89 | <0.001 |
| HbA_1c_>7% , n (%) | 1783 (80.9) | 1622 (78.0) | 0.018 |
| Total cholesterol, mmol/L | 5.66±1.39 | 4.48±1.02 | <0.001 |
| HDL-cholesterol, mmol/L | 1.17±0.29 | 1.07±0.26 | <0.001 |
| Triglyceride, mmol/L | 1.81 (1.29,2.73) | 1.48 (1.05,2.11) | <0.001 |
| LDL-cholesterol, mmol/L | 3.78±0.99 | 2.94±0.81 | <0.001 |
| Uric acid, umol/L | 331.01±94.47 | 331.03±93.01 | 0.980 |
| Indirect bilirubin, umol/L | 7.31±2.46 | 12.59±4.51 | <0.001 |
| Direct bilirubin, umol/L | 2.58±1.04 | 4.35±1.96 | <0.001 |
| Total bilirubin, umol/L | 9.89±2.66 | 16.94±5.52 | <0.001 |
| TBIL/TC ratio, % | 0.18±0.05 | 0.39±0.15 | <0.001 |
| GGT, U/L | 34.76±35.91 | 37.64±39.73 | 0.013 |
| AST, U/L | 21.30±13.64 | 23.63±14.88 | <0.001 |
| ALT, U/L | 23.00±20.18 | 27.60±23.17 | <0.001 |
| Baseline UACR, mg/g | 12.29 (6.28,49.68) | 9.47 (5.54,26.58) | <0.001 |
| Baseline eGFR, mL/min/1.73 m^2^ | 93.78±23.24 | 98.26±18.34 | <0.001 |
| Statins use, n (%) | 1484 (67.4) | 1093 (52.6) | <0.001 |
| ACEi/ARBs use, n (%) | 1341(60.9) | 1155 (55.6) | <0.001 |
| Insuline use, n (%) | 1341 (60.9) | 1155 (55.6) | <0.001 |
| SGLT2i use, n (%) | 423 (19.2) | 346 (16.6) | 0.029 |
| GLP-1RA use, n (%) | 215 (9.8) | 223 (10.7) | 0.297 |

Data are expressed as mean ± SD, median (IQR), or n (%). Abbreviations as Table S1.

^a^ participants with available smoking data at baseline (former/current or never) (n=3743)

^b^ participants with available alcohol consumption status data at baseline (former/current or never) (n=3674)

^c^ participants with history of CVD at baseline (n= 3024)

^d^ participants with hypertension at baseline (n= 3024)

Table S3 Univariate Cox regression analysis for CKD progression

| Variables | HR | 95% CI | *P* value |
| --- | --- | --- | --- |
| Male, n (%) | 1.11 | 0.93-1.32 | 0.235 |
| Age, years | 1.03 | 1.02-1.04 | <0.001 |
| Diabetic duration, years | 1.04 | 1.03-1.05 | <0.001 |
| Smoking, n (%) ^a^ | 1.17 | 0.97-1.42 | 0.106 |
| Alcohol consumption, n (%) ^b^ | 0.94 | 0.77-1.14 | 0.510 |
| History of CVD, n (%) ^c^ | 1.22 | 0.97-1.54 | 0.092 |
| BMI, kg/m^2^ | 1.00 | 0.99-1.01 | 0.803 |
| Systolic BP, mmHg | 1.02 | 1.02-1.03 | <0.001 |
| HbA_1c_,% | 1.08 | 1.04-1.13 | <0.001 |
| HDL-cholesterol, mmol/L | 1.26 | 0.93-1.71 | 0.134 |
| Log triglyceride, mmol/L | 2.08 | 1.53-2.84 | <0.001 |
| LDL-cholesterol, mmol/L | 1.29 | 1.19-1.41 | <0.001 |
| GGT, U/L | 1.00 | 1.00-1.00 | 0.771 |
| AST, U/L | 1.00 | 0.99-1.00 | 0.314 |
| ALT, U/L | 0.99 | 0.98-0.99 | <0.001 |
| TBIL/TC ratio, % | 0.03 | 0.01-0.07 | <0.001 |
| Log UACR baseline, mg/g | 3.49 | 3.17-3.84 | <0.001 |
| Statins use, n (%) | 1.42 | 1.19-1.71 | <0.001 |
| ACEi/ARBs use, n (%) | 1.73 | 1.46-2.06 | <0.001 |
| Insuline use, n (%) | 2.02 | 1.66-2.46 | <0.001 |
| GLP-1RA use, n (%) | 0.81 | 0.59-1.12 | 0.204 |

*P* values in bold are < 0.05.

Abbreviations as Table S1.

^a^ participants with available smoking data at baseline (former/current or never) (n=3743)

^b^ participants with available alcohol consumption status data at baseline (former/current or never) (n=3674)

^c^ participants with history of CVD at baseline (n= 3024)

Table S4 Association of TBIL/TC ratio with risk for CKD progression stratifying by albuminuria category (discovery cohort)

|  | UACR < 30 mg/g | UACR 30-300 mg/g | UACR ≥ 300 mg/g |
| --- | --- | --- | --- |
| TBIL/TC ratio | HR (95% CI) *P* value | HR (95% CI) *P* value | HR (95% CI) *P* value |
| Continuous (per unit) | 0.66 (0.21, 2.04) 0.466 | 0.15 (0.02, 1.07) 0.058 | 0.04 (0.01, 0.26) 0.001 |
| Quartiles (%) |  |  |  |
| Q1 (≤0.18) | Reference - | Reference - | Reference - |
| Q2 (0.18-0.25) | 0.79 (0.54, 1.15) 0.219 | 0.54 (0.33, 0.87) 0.011 | 0.80 (0.51, 1.25) 0.328 |
| Q3 (0.25-0.34) | 0.80 (0.53, 1.20) 0.273 | 0.42 (0.24, 0.73) 0.002 | 0.55 (0.32, 0.95) 0.031 |
| Q4 (>0.34) | 0.85 (0.54, 1.33) 0.464 | 0.45 (0.24, 0.84) 0.012 | 0.36 (0.19, 0.70) 0.002 |
| Reference point (%) |  |  |  |
| Low TBIL/TC ratio (≤0.25) | Reference - | Reference - | Reference - |
| High TBIL/TC ratio (>0.25) | 0.95 (0.70, 1.28) 0.721 | 0.62 (0.41, 0.96) 0.031 | 0.51 (0.33, 0.78) 0.002 |

Multivariate model adjusted for variables with *P* value < 0.05 in univariate analysis plus sex and BMI, including sex, age, BMI, systolic blood pressure, glycemic control, insulin treatment (yes or no), use of statin (yes or no), use of ACE inhibitors or angiotensin receptor blockers (ACEi/ARBs) (yes or no), logarithmically transformed triglycerides, duration of diabetes, LDL-cholesterol, ALT and logarithmically transformed UACR.

Table S5 Association of TBIL/TC ratio with risk for CKD progression stratifying by CKD status (discovery cohort)

|  | eGFR < 60 ml/min/1.73m^2^ | eGFR ≥ 60ml/min/1.73m^2^ |
| --- | --- | --- |
| TBIL/TC ratio | HR (95% CI) *P* value | HR (95% CI) *P* value |
| Continuous (per unit) | 0.26 (0.10, 0.70) 0.007 | 0.04 (0.00, 0.39) 0.006 |
| Quartiles (%) |  |  |
| Q1 (≤0.18) | Reference - | Reference - |
| Q2 (0.18–0.25) | 0.69 (0.52, 0.90) 0.007 | 0.44 (0.24, 0.79) 0.006 |
| Q3 (0.25–0.34) | 0.59 (0.44, 0.80) 0.001 | 0.50 (0.26, 0.97) 0.040 |
| Q4 (>0.34) | 0.61 (0.43, 0.86) 0.005 | 0.44 (0.22, 0.90) 0.025 |
| Reference point (%) |  |  |
| Low TBIL/TC ratio (≤0.25) | Reference - | Reference - |
| High TBIL/TC ratio (>0.25) | 0.75 (0.59, 0.95) 0.015 | 0.66 (0.40, 1.09) 0.101 |

Multivariate model adjusted for variables with *P* value < 0.05 in univariate analysis plus sex and BMI, including sex, age, BMI, systolic blood pressure, glycemic control, insulin treatment (yes or no), use of statin (yes or no), use of ACE inhibitors or angiotensin receptor blockers (ACEi/ARBs) (yes or no), logarithmically transformed triglycerides, duration of diabetes, LDL-cholesterol, ALT and logarithmically transformed UACR.

Table S6 Sensitivity analyses to investigate the association between TBIL/TC ratio and CKD progression (discovery cohort)

|  |  | Continuous variable | | Binary variable | | | |
| --- | --- | --- | --- | --- | --- | --- | --- |
|  |  |  |  | Low  TBIL/TC ratio(≤0.25%） |  | High  TBIL/TC  ratio (>0.25%） |  |
|  | Events/Total | HR (95% CI) | *P* value | HR (95% CI) | *P* value | HR (95% CI) | *P* value |
| Model further adjusted baseline eGFR | 522/4282 (12.2) | 0.24 (0.10, 0.58) | 0.001 | Reference | - | 0.78 (0.63, 0.96) | 0.017 |
| Model further adjusted SGLT2i and GLP-1RA use | 522/4282 (12.2) | 0.17(0.07, 0.42) | <0.001 | Reference | - | 0.73 (0.60, 0.90) | 0.003 |
| Model further adjusted smoking status ^a^ | 431/3743 (11.5) | 0.18 (0.07, 0.47) | <0.001 | Reference | - | 0.71 (0.57, 0.89) | 0.003 |
| Model further adjusted alcohol consumption ^b^ | 424/3674 (11.5) | 0.17 (0.06, 0.45) | <0.001 | Reference | - | 0.70 (0.56, 0.88) | 0.002 |
| Model further adjusted history of CVD and hypertension ^c^ | 753/3024  (24.9) and 1713/3024  (56.6) | 0.16 (0.06, 0.49) | 0.001 | Reference | - | 0.68 (0.52, 0.88) | 0.003 |
| No SGLT2i and GLP-1A use at baseline ^d^ | 393/3208 (12.3) | 0.14 (0.05, 0.40) | <0.001 | Reference | - | 0.79 (0.62, 1.00) | 0.047 |
| Model further adjusted uric acid | 522/4282 (12.2) | 0.17 (0.07, 0.41) | <0.001 | Reference | - | 0.74 (0.60, 0.91) | 0.004 |

Basic multivariate model adjusted for variables with *P* value < 0.05 in univariate analysis plus sex and BMI, including sex, age, BMI, systolic blood pressure, glycemic control, insulin treatment (yes or no), use of statin (yes or no), use of ACE inhibitors or angiotensin receptor blockers (ACEi/ARBs) (yes or no), logarithmically transformed triglycerides, duration of diabetes, LDL-cholesterol, ALT and logarithmically transformed UACR.

^a^ participants with available smoking data at baseline (former/current or never) (n=3743)

^b^ participants with available alcohol consumption status data at baseline (former/current or never) (n=3674)

^c^ participants with available history of CVD and hypertension data at baseline (n=3024)

^d^ participants without use of SGLT2i and GLP-1RA at baseline (n=3208)

Table S7 Comparison of baseline characteristics of the discovery and validation cohorts

| Variable | Discovery cohort (n=4282) | Validation cohort (n=758) | *P* value |
| --- | --- | --- | --- |
| Male, n (%) | 2366 (55.3) | 446 (58.8) | 0.067 |
| Age, years | 56.3±12.1 | 55.9±12.0 | 0.446 |
| Diabetic duration, years | 10.33±7.71 | 9.14±7.70 | <0.001 |
| Smoking, n (%) ^a^ | 1483 (39.6) (n=3743) | 316 (44.6) (n=708) | 0.013 |
| Alcohol consumption, n (%) ^b^ | 1372 (37.3) (n=3674) | 284 (41.1) (n=691) | 0.062 |
| History of CVD, n (%) ^c^ | 753 (24.9) (n=3024) | 103 (21.3) (n=484) | 0.085 |
| Hypertension, n (%) ^d^ | 1713 (56.6) (n=3024) | 266 (55.0) (n=484) | 0.487 |
| BMI, kg/m^2^ | 27.18±10.74 | 26.95±4.17 | 0.599 |
| Diastolic BP, mmHg | 80.90±10.62 | 82.19±11.50 | 0.004 |
| Systolic BP, mmHg | 135.01±18.07 | 135.57±17.21 | 0.429 |
| HbA1c,% | 8.76±1.97 | 8.66±2.05 | 0.238 |
| HbA1c>7% , n (%) | 3405 (79.5) | 595 (78.5) | 0.521 |
| Total cholesterol, mmol/L | 5.09±1.36 | 5.23±1.48 | 0.007 |
| HDL-cholesterol, mmol/L | 1.12±0.28 | 1.15±0.27 | 0.013 |
| Triglyceride, mmol/L | 1.65 (1.16, 2.42) | 1.67 (1.16, 2.44) | 0.653 |
| LDL-cholesterol, mmol/L | 3.37±1.00 | 3.51±1.04 | <0.001 |
| Uric acid, umol/L | 331.06±93.76 | 342.05±97.91 | 0.003 |
| Indirect bilirubin, umol/L | 9.87±4.47 | 10.57±4.66 | <0.001 |
| Direct bilirubin, umol/L | 3.44±1.79 | 4.06±1.98 | <0.001 |
| Total bilirubin, umol/L | 13.31±5.56 | 14.63±6.33 | <0.001 |
| TBIL/TC ratio, % | 0.28±0.15 | 0.30±0.16 | 0.003 |
| GGT, U/L | 36.16±37.84 | 38.89±58.85 | 0.096 |
| AST, U/L | 22.44±14.30 | 25.02±15.88 | <0.001 |
| ALT, U/L | 25.23±21.81 | 27.51±22.40 | 0.008 |
| Baseline UACR, mg/g | 10.77 (5.92,36.16) | 10.90 (5.32,53.24) | 0.712 |
| Baseline eGFR, mL/min/1.73 m^2^ | 97.96±21.12 | 97.79±22.14 | 0.029 |
| Statins use, n (%) | 2577 (60.2) | 485 (64.0) | 0.048 |
| ACEi/ARBs use, n (%) | 1569 (36.6) | 414 (54.6) | <0.001 |
| Insuline use, n (%) | 2496 (58.3) | 358 (47.2) | <0.001 |
| SGLT2i use, n (%) | 769 (18.0) | 370 (48.8) | <0.001 |
| GLP-1RA use, n (%) | 438 (10.2) | 124 (16.4) | <0.001 |

Data are expressed as mean ± SD, median (IQR), or n (%). Abbreviations as Table S1.

^a,b,c,d^ data available at baseline.

Table S8 HRs (95% CIs) for progression of CKD based on the TBIL/TC ratio (validation cohort)

|  |  | Model 1 | Model 2 | Model 3 |
| --- | --- | --- | --- | --- |
| TBIL/TC ratio | Events/Total | HR (95% CI) *P* value | HR (95% CI) *P* value | HR (95% CI) *P* value |
| Continuous(per unit) | 65/758 (8.6) | - | - | 0.07(0.01,0.88) 0.039 |
| Quartiles (%) |  |  |  |  |
| Q1 (≤0.20) | 34/213 (16.0) | Reference - | Reference - | Reference - |
| Q2 (0.20-0.27) | 13/188 (6.9) | 0.41 (0.22,0.78) 0.007 | 0.38 (0.20,0.73) 0.003 | 0.55 (0.27,1.12) 0.099 |
| Q3 (0.27-0.37) | 9/182 (4.9) | 0.25 (0.12,0.53) <0.001 | 0.23 (0.11,0.48) <0.001 | 0.62 (0.26,1.47) 0.277 |
| Q4 (>0.37) | 9/175 (5.1) | 0.28 (0.14,0.59) 0.001 | 0.23 (0.11,0.48) <0.001 | 0.37(0.15,0.93) 0.035 |

Model 1crude

Model 2 adjusted by sex and age

Model 3 adjusted for variables with *P* value < 0.05 in univariate analysis plus sex and BMI, including sex, age, BMI, systolic blood pressure, glycemic control, insulin treatment (yes or no), use of statin (yes or no), use of ACE inhibitors or angiotensin receptor blockers (ACEi/ARBs) (yes or no), logarithmically transformed triglycerides, duration of diabetes, LDL-cholesterol, ALT and logarithmically transformed UACR.

Figure S1 Flow chart of the validation cohort


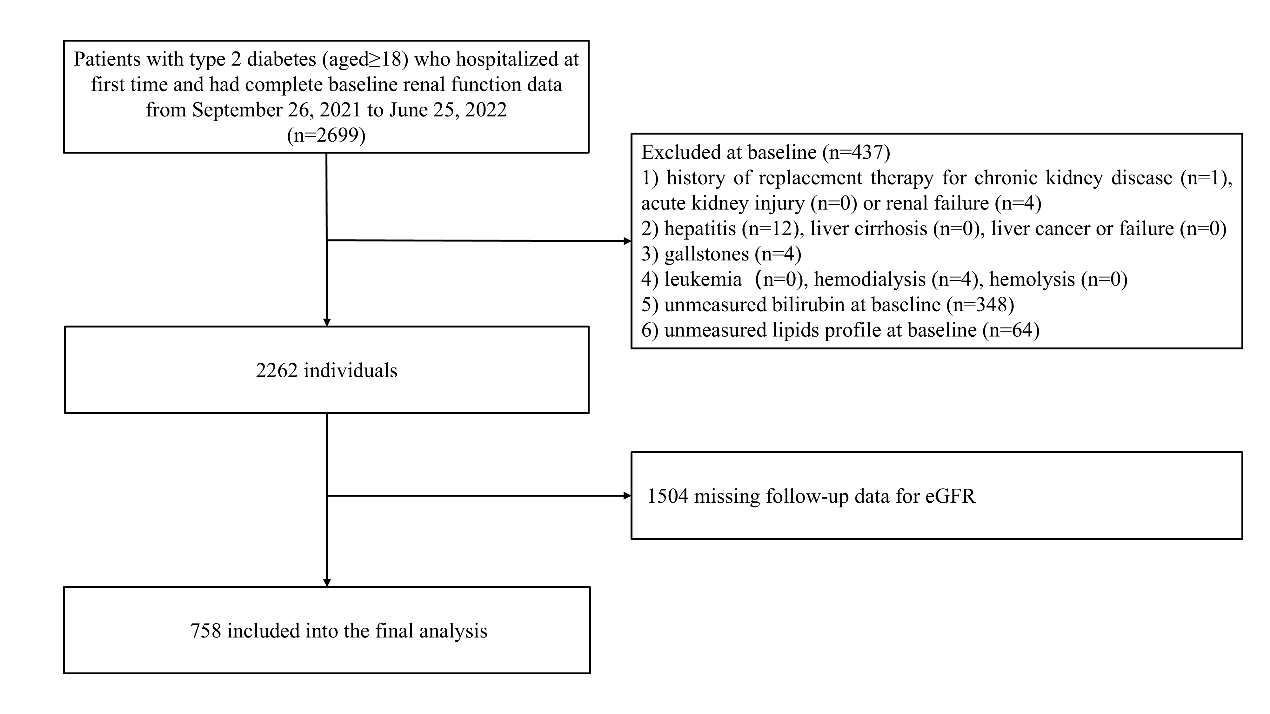


Figure S2 The association between the TBIL/TC ratio and CKD progression with 3-knot (A) and 5-knot (B) of RCS model (discovery cohort)


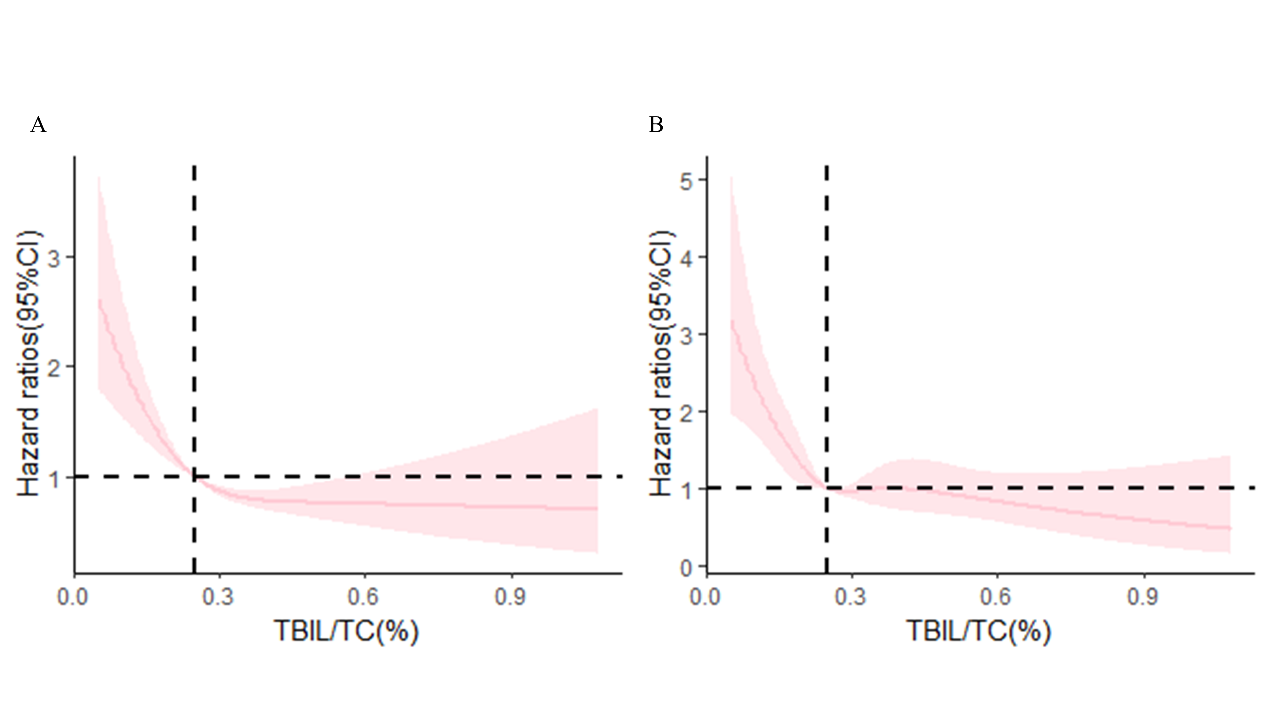


Note: The following covariates at baseline were incorporated into the model: sex, age, BMI, systolic blood pressure, glycemic control, insulin treatment (yes or no), use of statin (yes or no), use of ACE inhibitors or angiotensin receptor blockers (ACEi/ARBs) (yes or no), logarithmically transformed triglycerides, duration of diabetes, LDL-cholesterol, ALT and logarithmically transformed UACR.
